# 20902 Elucidation of Cardioprotective Mechanisms via Human Models of Chemotherapy-Induced Cardiotoxicity

**DOI:** 10.1017/cts.2021.404

**Published:** 2021-03-30

**Authors:** Nnamdi Uche, Qiang Dai, Shuping Lai, Ivor Benjamin

**Affiliations:** Medical College of Wisconsin

## Abstract

ABSTRACT IMPACT: This work should provide further insights to mechanisms of the negative consequences of chemotherapy drugs, specifically in the cardiovascular system. OBJECTIVES/GOALS: Cardiotoxicity remains a safety concern in the development or utilization of chemotherapeutics largely due to the gap in knowledge of the mechanisms of toxicity. The pathophysiology of this cardiotoxicity has not been fully elucidated but data from our lab as well as other recent studies hint toward implications of mitochondrial (mito) biogenesis. METHODS/STUDY POPULATION: Prophylactic use of the beta-blocker carvedilol as well as the ACE inhibitor enalapril have been shown to inhibit the development of anthracycline-induced toxicity, but the mechanism of this cardio-protection remains elusive. To explore this, human stem cell-derived cardiomyocytes and endothelial cells will be either treated with the anthracycline doxorubicin or pretreated with carvedilol or enalapril followed by doxorubicin treatment before cellular lysates are harvested. Western blotting and qPCR will be performed to determine the expression of mito biogenesis markers including Nrf1, TFAM and the master regulator of mito biogenesis, PGC-1α. RESULTS/ANTICIPATED RESULTS: We anticipate that doxorubicin treatment alone will result in decreased expression of the mito biogenesis markers Nrf1, TFAM and PGC-1α and that pretreatment with either carvedilol and/or enalapril prior to doxorubicin treatment will either prevent or reverse this. DISCUSSION/SIGNIFICANCE OF FINDINGS: Doxorubicin’s role in causing mitochondrial dysfunction as well as suppression of biogenesis has already been established. Ideally, generation of new mitochondria would offset the occurrence of dysfunctional mitochondria. Confirming carvedilol/enalapril’s involvement with mito biogenesis would provide a mechanism of cardio-protection.

